# The anti-tumor effects of evodiamine on oral squamous cell carcinoma (OSCC) through regulating advanced glycation end products (AGE) / receptor for advanced glycation end products (RAGE) pathway

**DOI:** 10.1080/21655979.2021.1972082

**Published:** 2021-09-03

**Authors:** Liuyang Ren, Ying Lou, Mingyu Sun

**Affiliations:** a Department of Stomatology, Henan Provincial People’s Hospital, People’s Hospital of Zhengzhou University, People’s Hospital of Henan University, Zhengzhou, China; b Department of Stomatology, Union Hospital, Huazhong University of Science and Technology, Wuhan, P. R. China

**Keywords:** Evodiamine, oral squamous cell carcinoma, advanced glycation end products, receptor for advanced glycation end products, high-mobility group box 1

## Abstract

Evodiamine (EVO) is emerging as a novel anti-tumor drug, which is involved in the inhibition of cell proliferation and apoptosis. High-Mobility Group Box 1 (HMGB1)/RAGE is involved in invasive behavior of OSCC cells and angiogenesis. In this study, we evaluated the potential of EVO in OSCC *in vitro* and *in vivo*. We found that RAGE silencing suppressed HSC-4 cell proliferation and invasion, and tube formation of HUVEC. EVO showed marked inhibitory effects on the malignant behaviors of HSC-4 cells in a dose-dependent manner. Further experiments revealed that the RAGE overexpression was able to markedly block the effects of EVO on cell proliferation and invasion, and tube formation. By analyzing the expression of High-Mobility Group Box 1 (HMGB1) and RAGE in HSC-4 cells, the result showed that EVO slightly reduced HMBG1 levels and dramatically decreased RAGE levels, while RAGE overexpression did have no marked influences on HMBG1 levels. The anti-tumor effects of EVO were further confirmed in mouse oral squamous cell carcinoma xenograft models. Remarkable anti-tumor effects of EVO were also demonstrated, as presented by reduced tumor size and levels of HMBG1 and RAGE in tumor tissue of mouse oral squamous cell carcinoma xenograft models. The results demonstrated that EVO has a direct binding effect on HMGB1, but it may be involved in degrading the protein. More importantly, it can reduce the activity of RAGE pathway by affecting the binding between HMBG1 and RAGE. To conclude, EVO inhibited proliferation, invasion and angiogenesis of OSCC through affecting the downstream signal transduction system of AGE/RAGE by targeting RAGE.

## Introduction

OSCC accounts for the eighth most common cancer in the world and leads to serious health threat with a 5-year survival rate of less than 60% [1]. The current therapeutic methods of OSCC involve mainly comprehensive surgical operations combined with other adjuvant treatment methods, including systemic chemotherapy, local radiation, biological therapy, etc. [2–4]. Squamous cell carcinoma (SCC) expressed HMGB1 and RAGE and their interaction was involved in the migration of SCC cells [5]. Furthermore, HMGB1 produced by OSCC was able to lead to bone destruction through RAGE [6]. Studies have suggested that RAGE pathway, which can be activated in OSCC, is significantly correlated with the differentiation degree of OSCC. Further pieces of evidence also linked the highly expressed RAGE pathway to cell proliferation, invasion and angiogenesis activity [7–10].

Traditional Chinese medicine (TCM) related studies on reducing the expression level of RAGE have found that many ‘acid’ drugs have such effects on decreasing RAGE levels [11,12]. It was predicted by Bioinformatics Analysis Tool for Molecular mechanism of Traditional Chinese Medicine (BATMAN-TCM) database that the active substance EVO of *Edodia rutaecarpa* could act on RAGE or the main ligands of RAGE. Among them, the active substance EVO of Edodia rutaecarpa has been reported in many studies to inhibit the proliferation of various tumors and promote cell apoptosis [13–15]. EVO is the main alkaloid component of Evodia, and its molecular formula is C19H17N3O. Molecular docking with Swissdock showed that EVO could bind to HMGB1, the most common ligand that activates RAGE in OSCC [5,8,16]. In addition, EVO has potential effects on the treatment of oral inflammation caused by radiotherapy and chemotherapy [17]. It is still unclear about the effects of EVO on OSCC and the mechanism events involved. Our investigations wound shed light on the mechanism of EVO in OSCC and provide molecular basis to predict an effect of EVO on tumor growth in OSCC.

Based on the above background, we speculated that EVO might regulate the functional phenotypes of OSCC cells by affecting the RAGE signaling pathway, and thus the experiments were designed to confirm our hypothesis and was expected to elucidate the molecular events behind the effects of EVO on tumor growth in OSCC.

## Method

### Cell line

A-253, HSC-4, CAL-27, SCC-4 and HUVEC cell lines (American Type Culture Collection, USA) were cultured in DMEM high glucose medium (Gibco, USA) containing 10% fetal bovine serum and 1% penicillin-streptomycin, and the medium was changed once every 2 days. The cell line was digested and passed when the cell confluence degree arrived 80%~90%. HSC-4 cells were treated with different concentration of EVO (1, 2, 4, 8 and 16 µmol/L) for 24, 48 or 72 h for evaluation of cell proliferation.

### Quantitative reverse transcription PCR (RT-qPCR)

Total RNA was extracted from OSCC cell line according to Trizol reagent instructions (Sigma-Aldrich). After extraction, the RNA concentration was tested by UV spectrophotometer and the completeness of RNA was detected by gel electrophoresis. The cDNA was synthesized according to the instructions of the reverse transcription kit (Roche). SYBR® Premix Ex Taq II (2×) 10 µL (TAKARA) was used for PCR amplification in PCR instrument Mx3000P (Agilent, USA). The PCR conditions consisted of initial denaturation for 2 min at 94°C, followed by cycles of 40, denaturation for 20 s at 94°C, annealing for 20 s at 58°C and extension for 20 s at 72°C. GAPDH mRNA was used as internal reference. 2^−ΔΔCt^ method was used to calculate the expression level of target genes [18]. The primer sequences are as following: RAGE, Forward: 5ʹ-GTGTCCTTCCCAACGGCTC-3ʹ, Reverse: 5ʹ-ATTGCCTGGCACCGGAAAA-3ʹ. HMGB1, Forward: 5ʹ-TATGGCAAAAGCGGACAAGG-3ʹ, Reverse: 5ʹ-CTTCGCAACATCACCAATGGA-3ʹ. GAPDH Forward: 5ʹ-TGTTCGTCATGGGTGTGAAC-3ʹ, Reverse: 5ʹ-ATGGCATGGACTGTGGTCAT-3ʹ.

### Plasmid transfection

The cultured HSC-4 or HUVEC cells were divided into different groups according to experimental conditions. The plasmids silencing RAGE (KD-RAGE) or overexpressing RAGE (Ov-RAGE) and their control plasmids were constructed and purchased from GenePharma (Shanghai, China). The cells were transfected with KD-RAGE or Ov-RAGE using Lipofectamine® 2000 according to manufacturer’s guidance (Thermo Fisher Scientific). After 12 h, cells were treated with EVO 4 µmol/L for the next experiments.

### Cell counting Kit-8 (CCK8) assay

Cell suspension (about 1 × 10^5^ cells) was added to 96-well plates, and a negative control was set. The cells were incubated overnight in 37°C with 5% CO_2_. The absorbance at 450 nm was detected after CCK8 solution (GlpBio, USA) was added for incubation for 24, 28 or 72 h, respectively.

### Transwell assay

The Matrigel glue was diluted with DMEM medium at the ratio of 1:10. Then, 100 μL Matrigel was added into the upper chamber of transwell chamber (Corning, USA). The cells were resuspended in DMEM medium, adjusted to 3 × 10^5^ cells/mL, and 100 μl of the cells was added into the upper chamber. The lower chamber was added with DMEM medium 600 μL containing 10% serum. The cells were stained with 0.1% crystal violet for 20 min at room temperature according to Transwell instructions. After staining, three fields were selected under a light microscope to count transmembrane cells and calculate the number of invaded cells.

### Tube formation assay

50 μL Matrigel was evenly spread on the bottom of the 96-well plate in each well and placed in a cell incubator at 37°C with 5% CO_2_ to solidify the Matrigel. We used trypsin to digest and collected transfected HUVECs. 1 × 10^4^ cells were added to each well. DMEM medium containing 100 μL 10% FBS and EVO was used to culture cells for 24 h. The formation of vascular lumen was observed under an inverted microscope (CKX53, Olympus, Japan).

### Western blotting

The cells or tumor tissue were collected and lysed using RIPA lysis buffer. The supernatant was collected after centrifugation. The protein sample (20 µg) was uploaded and separated through 10% SDS-PAGE gel electrophoresis and then cells were transferred to PVDF membrane. TBST containing 5% BSA was added and used to block protein at room temperature for 1 h. Next, the protein bands were incubated with the primary antibodies (HMBG1, VEGF: 1:10,000; RAGE, c-Jun, NF-κB, MMP-2, IL-6, TNF-α, 1:1000) overnight at 4°C, followed by the incubation with secondary antibody (Goat Anti-Rabbit, 1:10,000, Abcam, England) for 2 h at room temperature. Liquid A of chemiluminescence reagent was mixed with liquid B by the ratio of 1:1; then, protein bands were evenly added to develop color. The relative protein expression was the ratio of the gray value between the target band and the internal reference band (GAPDH was used as internal reference). The relative expression levels of target proteins were analyzed using Image J software 1.46 r (National Institutes of Health).

### Tumor-bearing mice experiment

Twelve BALB/c Nude mice (6 weeks, 18–19 g, female) were purchased from the Charles River (Beijing, China). HSC-4 cell suspension (1 × 10^6^/10 μL) with empty vectors (OverExp-vector) or plasmids overexpressing RAGE (Ov-RAGE) transfection was prepared and injected into right forelimb armpit. After 1 day, the mice were administrated with EVO (3 mg/kg) or normal saline by gavage once a day, for 21 days.

The animals were sacrificed on the next day after the last administration, and the tumor tissues were collected (partly −20°C was frozen for testing, and partly fixed in 4% paraformaldehyde solution). The study was approved by the ethical committee of Tongji Medical College.

### Hemotoxylin and eosin staining (HE) staining

Slices prepared were soaked in xylene for 10 min. Then, gradient dehydration was carried out with alcohol, rinsed and soaked in hematoxylin (Sangon Biotech). The running water was used to rinse for 2 min at a time and then slices were soaked in alcohol containing 1% hydrochloric acid. After rinsing, stain was performed with lithium carbonate. It was soaked in ethanol, stained with eosin, and then treated with alcohol and soaked in xylene for 2 min. The sections were observed under an inverted microscope (Olympus).

### Statistical analysis

All data were statistically analyzed using GraphPad Prism 8.0 software. One-way ANOVA test was used for comparison among groups, followed by tukey's test between two groups. p < 0.05 indicates that the difference is statistically significant.

## Results

### RAGE silencing suppressed the proliferation and migration of HSC-4 cells

**Figure 1.**
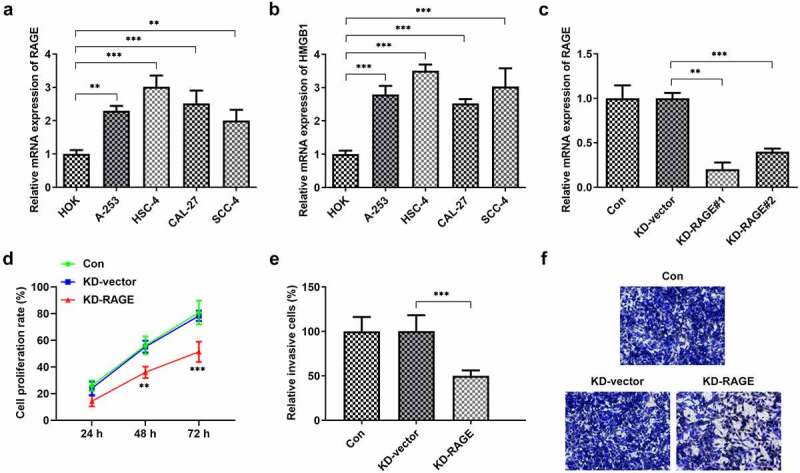
RAGE silencing reduced the proliferation and invasion of HSC-4 cells. (a-b) The expression levels of RAGE and HMGB1 were detected in different OSCC cell lines through RT-qPCR. (c) RAGE expression was reduced after KD-RAGE#1 or KD-RAGE#2 transfection. (d) The cells proliferation was analyzed after 24, 48 h or 72 h of transfection with KD-RAGE#1. (e-f) The cell invasion abilities were detected through transwell assay. **p < 0.01, ***p < 0.001

To study the roles of HMGB1/RAGE in OSCC, qPCR analysis of RAGE and HMGB1 was performed in several OSCC lines. Compared to the HOK group, the levels of RAGE and HMGB1 mRNA were markedly increased in OSCC lines (Figure 1a-b). RAGE was knocked out to study its effect on OSCC. We used KD-RAGE#1 to perform further experiments because it could more efficiently silence RAGE1 than KD-RAGE#2 (Figure 1c). Next, we found that compared with the control group, RAGE silencing significantly suppressed the proliferation, invasion (Figure 1d-f).

### RAGE mediated the effects of EVO on tube formation of HUVEC cells and the proliferation and invasion of HSC-4 cells

**Figure 2.**
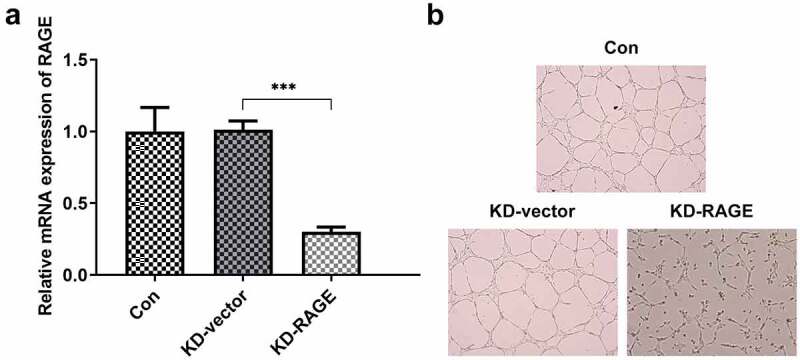
RAGE silencing resulted in the decrease of tube formation in HUVECs. (a) KD-RAGE transfection led to decreased expression of RAGE through RT-qPCR analysis in HSC-4 cells. (b) Effect of RAGE on HUVEC lumen formation ability. ***p < 0.001

**Figure 3.**
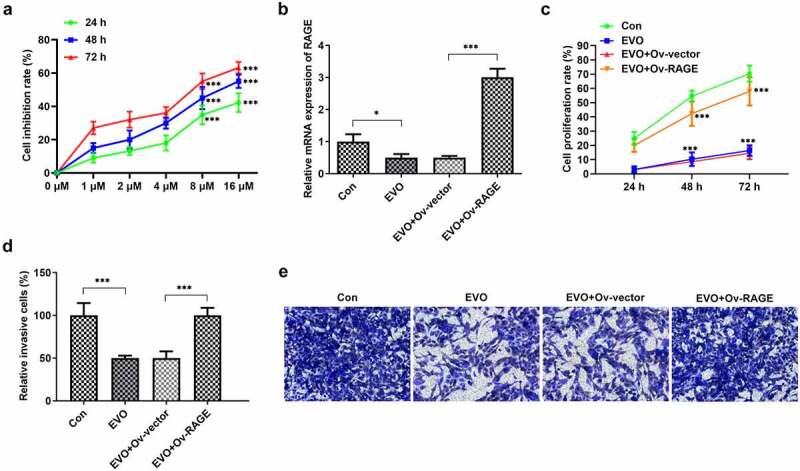
RAGE overexpression abolished the effects of EVO on proliferation and invasion in HSC-4 cells. (a) The proliferation levels of HSC-4 cells were detected by the treatment of 1, 2, 4, 8 or 16 µmol/L. (b) The detection of RAGE mRNA levels, (c) cell proliferation, (d–e) cell invasion when HSC-4 cells were treated with EVO or Ov-RAGE plasmid treatment. ***p < 0.001

**Figure 4.**
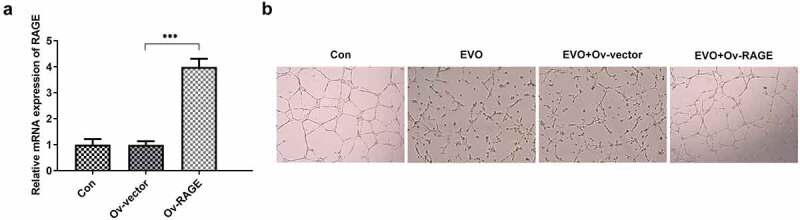
RAGE overexpression blunted the effects of EVO on tube formation in HUVEC cells. (a) Expression of RAGEs was increased when cells were cultured with RAGE overexpressing plasmids. (b) The tube formation abilities of HUVEC cells were analyzed through tuber formation assay. ***p < 0.001

To explore whether the mechanism of EVO was related to RAGE in OSCC, HUVEC cells and HSC-4 cells were employed. As shown in Supplementary material, evodia was predicted to have binding sites with HMGB1 by Swissdock, an online docking web server. Next, KD-RAGE plasmids were transfected into HSC-4 cells to induce decreased levels of RAGE mRNA in HUVEC cells (Figure 2a). Compared with the vector group, the number of luminal branch points in KD-RAGE was distinctly reduced (Figure 2b). Following incubation of EVO at different concentrations with HSC-4 cells for 24, 48 or 72 h, we could see from Figure 3a that at 4 µM, the cells were in a state of slight proliferation, and after 8 µM, the cell proliferation was lessened. IC50 of EVO at 24, 48 and 72 h was 5.77, 5.56 and 5.92, respectively. Therefore, EVO at the concentration of 4 µM was selected for subsequent experiments. In the next experiment, we wondered to know the role of RAGE following the treatment of EVO in HSC-4 cells. The results showed that RAGE mRNA levels were markedly reduced in HSC-4 cells with EVO treatment when compared with control cells and its overexpression significantly reversed the effects of EVO on suppressing cell proliferation and migration (Figure 3b-e). To further understand the role of RAGE in the tube formation of HUVEC cells, we used RAGE overexpressing plasmids to overexpress RAGE levels (Figure 4a). The number of luminal branch points was increased to close to normal levels when RAGE was overexpressed in EVO-treated cells (Figure 4b).

### *EVO suppressed HMGB1/RAGE pathway* in vitro *and* vivo

**Figure 5.**
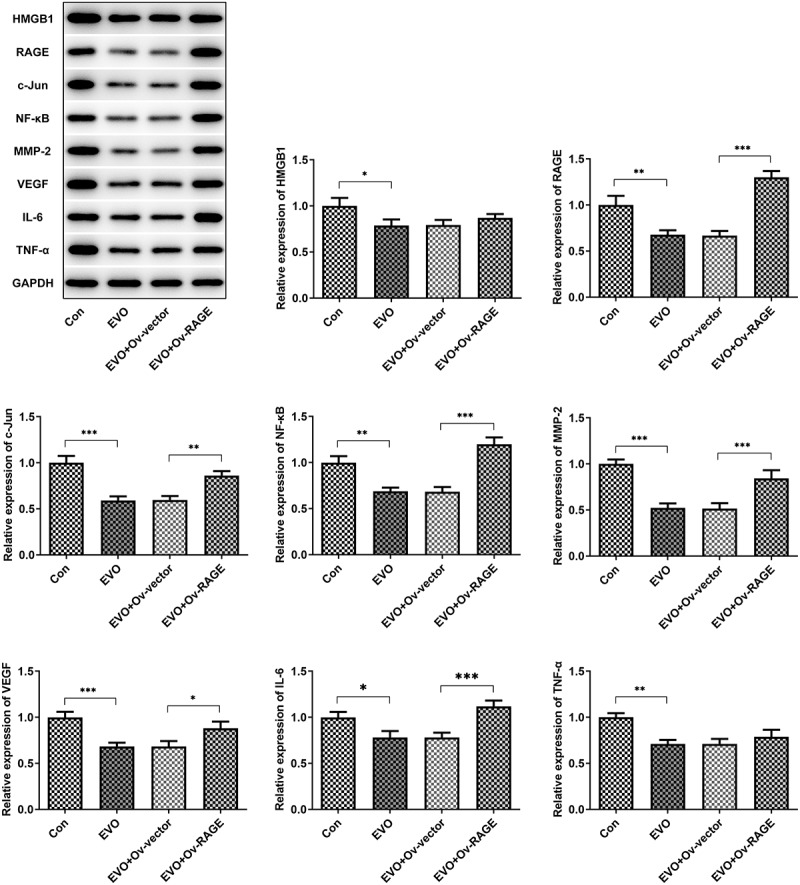
The analysis of HMBG1, RAGE, c-Jun, NF-κB, MMP-2, VEGF and IL-6 expression *in vitro* after EVO treatment or RAGE overexpression induction. *p < 0.05, **p < 0.01, ***p < 0.001

**Figure 6.**
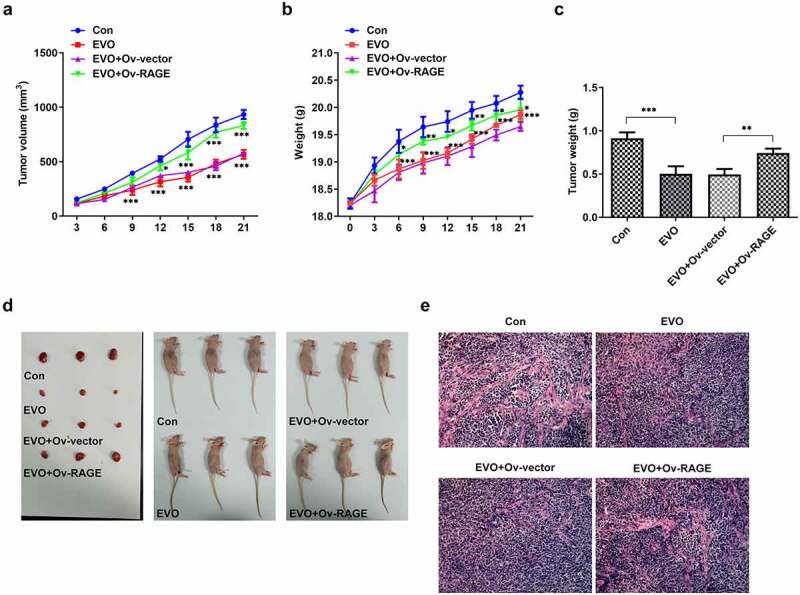
EVO administration suppressed tumor growth and vasculogenesis via RAGE. (a) The tumor volume, (b) mice weight, (c) tumor weight, (d) photos of mice and (e) Hemotoxylin and eosin staining. *p < 0.05, **p < 0.01, ***p < 0.001

**Figure 7.**
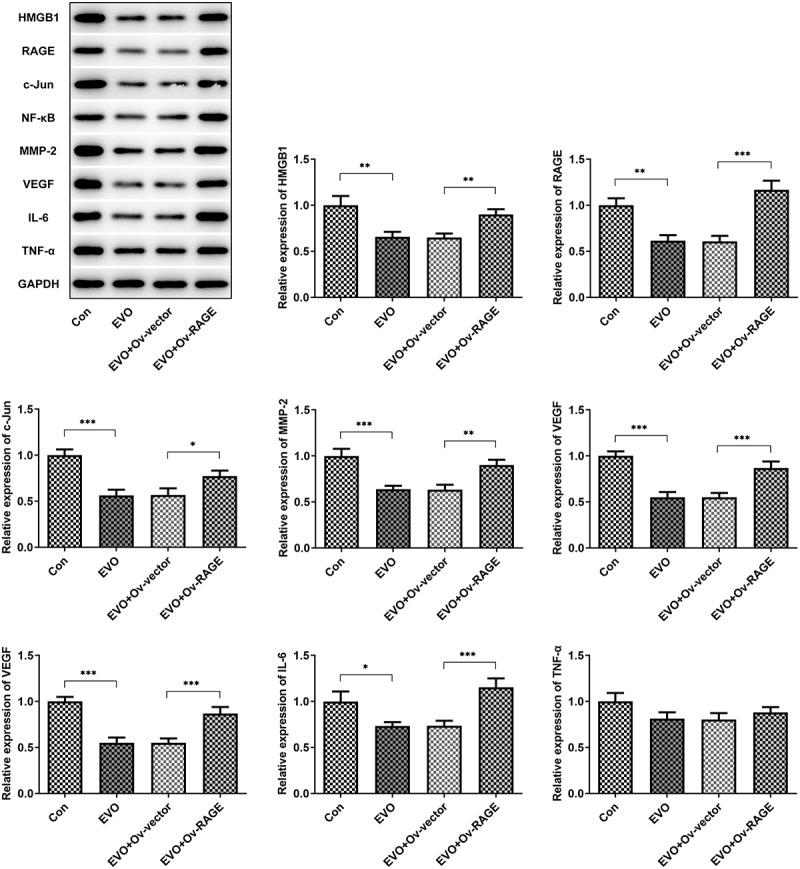
The levels of HMBG1, RAGE, c-Jun, NF-κB, MMP-2, VEGF and IL-6 were affected by EVO treatment, which was mediated by RAGE *in vivo*. *p < 0.05, **p < 0.01, ***p < 0.001

The effects of EVO on HMGB1/RAGE pathway were further evaluated in *in vitro* and *in vivo*. Studies have shown that RAGE is one of the receptors of HMGB1 and can bind to HMGB1 to play a pro-inflammatory role. VEGF, an angiogenetic factor, played a vital role in angiogenesis in OSCC and could be induced by HMGB1/RAGE axis [10]. In this process, the expression of HMGB1/RAGE, MMP2, VEGF and inflammatory factors in HSC-4 cells was detected by Western blotting method. The results showed that EVO could significantly reduce the levels of these factors. NF-κB signaling pathway was involved in the regulation of TNF-α, IL-6 and VEGF [19]. Therefore, NF-κB signaling pathway could be engaged in the effects of EVO on reducing the expression levels of these factors. The decreased HMGB1 and RAGE levels were observed after EVO treatment (Figure 5), which indicated that EVO could induce the degradation of HMGB1 through binding to it. More importantly, EVO could interfere with the binding effects of HMGB1 with RAGE to reduce the RAGE pathway activity. Subsequently, the decreased levels of RAGE were induced due to the positive feedback effect of RAGE pathway on RAGE. The transfected cell lines were subcutaneously injected into nude mice, and EVO was given by gavage for intervention. After 21 days, the tumor size was observed. The tumor volume and weight were significantly decreased after EVO treatment, which were significantly reversed upon RAGE overexpression (Figure 6a-c). The tumor tissue and the mice were photographed and presented in Figure 6d. Significant decreases in the density of vascular structure and the thickness of vessel wall were found in tumor treated with EVO, which were markedly reversed by RAGE overexpression (Figure 6e). To further confirm the results that EVO reduced cell proliferation, invasion and tube formation by HMBG1/RAGE axis, we analyzed RAGE signaling related proteins in the presence of EVO or RAGE overexpression (Figure 7). Intriguingly, treatment of cells with EVO and RAGE overexpression induction upregulated the levels of HMBG1, RAGE, c-Jun, NF-κB, MMP-2, VEGF and IL-6 compared with cells treated with EVO alone (Figure 7).

## Discussion

The major cause of morbidity and mortality in head and neck cancers occurs in OSCC patients. Current treatment for this disease, regardless of its capability of slightly improving the health status of patients, was found to easily trigger nonspecific cell death [20]. The cytotoxic effects of EVO against many cancers including colorectal cancer and non-small cell lung cancer have been reported, which were associated with the regulation of signaling pathway [21–23]. In initial experiments, the effects of different concentrations of EVO were investigated in the *in vitro* model and cell proliferation was found to be suppressed in a dose-dependent manner. Besides the reduced proliferation, we observed the suppression of invasion and tube formation by EVO in a dose-dependent manner *in vitro*. Furthermore, EVO administration suppressed tumor growth in mice injected HSC-4 cell suspension, suggesting that EVO has potential protective effects against OSCC.

RAGE is considered to be a predictor of relapse in patients with OSCC [24]. We used Swissdock database to conduct molecular docking and found that EVO has binding sites with HMGB1. By altering NF-κB activation and MMP levels, the invasion of OSCC cells by cigarette smoke extract was modulated, which was considered to be related to RAGE [25]. It was demonstrated that silencing of RAGE promoted HSC-4 cell proliferation and suppressed invasion of HSC-4 cell and tube formation of HUVECs, while RAGE overexpression abolished the effects of EVO on the above cell behaviors. Furthermore, *in vivo*, overexpression of RAGE abrogated the inhibitory effects of EVO on vascular structure and density. By analyzing the expression levels of proteins including RAGE, c-Jun, NF-κB, MMP-2, VEGF, IL-6 and TNF-α, the results showed that EVO treatment result in marked decreases, these effects of which were effectively abolished by the induction of RAGE overexpression. However, EVO slightly reduced HMGB1 levels and dramatically decreased RAGE levels, which implied that EVO could induce HMGB1 degradation and ameliorate RAGE activities to exert inhibitory effects on the above cell behaviors. Consistently, substantial evidence has revealed that HMGB1/RAGE axis was closely associated with the proliferation and invasion [26–28]. A study demonstrated that silencing of HMGB1 or RAGE resulted in the expression of NF-кB, while HMGB1 contributed to the activation of RAGE signaling pathways and NF-кB to facilitate certain malignant behaviors of cells, such as invasion and metastasis [29]. VEGF and MMP-2 are involved in the invasion of malignant cells to surrounding healthy tissue [30,31]. Besides, VEGF is implicated in regulating angiogenesis [32,33] and plays an important role in the metastasis of OSCC [34,35]. In view of this, the decreased invasion and angiogenesis by EVO mediated by RAGE could be related to the alteration of expression levels of VEGF and MMP-2. We conclude that EVO is able to suppress the proliferation and invasion of OSCC cells, together with the tube formation of HUVEC through HMGB1/RAGE signaling pathway.

## Conclusion

*In vitro and vivo* study found that EVO plays an effective role in suppressing tumor growth and angiogenesis, the role of which was confirmed to be related to HMGB1/RAGE axis. This research provides a basis for the future application of EVO in patients with OSCC and a novel sight for investigating the action mechanism of EVO.

## Limitation

Further investigations are required to reveal the events behind the involvemen of HMGB1/RAGE axis on the influences of EVO on inhibiting invasion and angiogenesis of oral cancer, as well as further experiments which are needed for the clinical application of EVO.

## Supplementary Material

Supplemental Material

## Highlights

1. RAGE regulated the proliferation and invasion of OSCC cells and tube formation.
2. HSC-4 cells displayed decreased proliferation and invasive behaviors in response to EVO, which was found to be related to HMGB1/RAGE axis.
3. EVO was able to suppress the expression levels of the tumor promoting factors and tumor growth by HMGB1/RAGE axis.
