# Supplementary figures and images for: The anti-tumor effects of evodiamine on oral squamous cell carcinoma (OSCC) through regulating advanced glycation end products (AGE) / receptor for advanced glycation end products (RAGE) pathway
Source: Bioengineered. 2021 Sep 3;12(1):5985–95. doi: 10.1080/21655979.2021.1972082 (PMC8806666; doi:10.1080/21655979.2021.1972082)

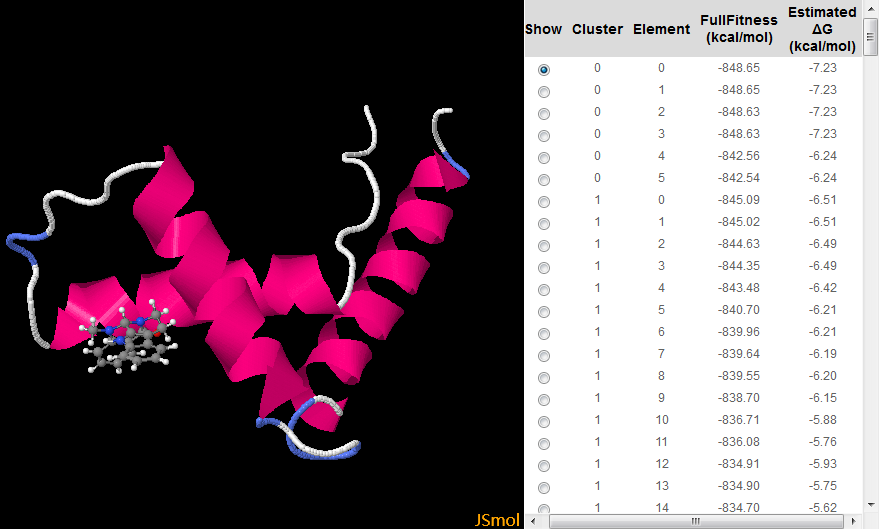

Supplement: Supplemental Material [file KBIE_A_1972082_SM3218.tif]
